# Electrical impedance tomography during spontaneous breathing trials and after extubation in critically ill patients at high risk for extubation failure: a multicenter observational study

**DOI:** 10.1186/s13613-019-0565-0

**Published:** 2019-08-13

**Authors:** Federico Longhini, Jessica Maugeri, Cristina Andreoni, Chiara Ronco, Andrea Bruni, Eugenio Garofalo, Corrado Pelaia, Camilla Cavicchi, Sergio Pintaudi, Paolo Navalesi

**Affiliations:** 1 Anesthesia and Intensive Care, Sant’Andrea Hospital, ASL VC, Vercelli, Italy; 2 Anesthesia and Intensive Care, “Garibaldi Centro” Hospital, ARNAS Garibaldi, Catania, Italy; 3 Anesthesia and Intensive Care, Infermi Hospital, AUSL Romagna, Rimini, Italy; 4 Anesthesia and Intensive Care Unit, University Hospital Mater Domini, Department of Medical and Surgical Sciences, Magna Graecia University, Viale Europa - Loc. Germaneto, 88100 Catanzaro, Italy

**Keywords:** Mechanical ventilation, Weaning, Spontaneous breathing trial, Post-extubation respiratory failure, Extubation failure, Electrical impedance tomography

## Abstract

**Background:**

This study aims to assess the changes in lung aeration and ventilation during the first spontaneous breathing trial (SBT) and after extubation in a population of patients at risk of extubation failure.

**Methods:**

We included 78 invasively ventilated patients eligible for their first SBT, conducted with low positive end-expiratory pressure (2 cm H_2_O) for 30 min. We acquired three 5-min electrical impedance tomography (EIT) records at baseline, soon after the beginning (SBT_0) and at the end (SBT_30) of SBT. In the case of SBT failure, ventilation was reinstituted; otherwise, the patient was extubated and two additional records were acquired soon after extubation (SB_0) and 30 min later (SB_30) during spontaneous breathing. Extubation failure was defined by the onset of post-extubation respiratory failure within 48 h after extubation. We computed the changes from baseline of end-expiratory lung impedance (∆EELI), tidal volume (∆Vt%), and the inhomogeneity index. Arterial blood was sampled for gas analysis. Data were compared between sub-groups stratified for SBT and extubation success/failure.

**Results:**

Compared to SBT success (*n* = 61), SBT failure (*n* = 17) showed a greater reduction in ∆EELI at SBT_0 (*p* < 0.001) and SBT_30 (*p* = 0.001) and a higher inhomogeneity index at baseline (*p* = 0.002), SBT_0 (*p* = 0.003) and SBT_30 (*p* = 0.005). RR/Vt was not different between groups at baseline but was significantly greater at SBT_0 and SBT_30 in SBT failures, compared to SBT successes (*p* < 0.001 for both). No differences in ∆Vt% and arterial blood gases were observed between SBT success and failure. The ∆Vt%, ∆EELI, inhomogeneity index and arterial blood gases were not different between patients with extubation success (*n* = 39) and failure (*n* = 22) (*p* > 0.05 for all comparisons).

**Conclusions:**

Compared to SBT success, SBT failure was characterized by more lung de-recruitment and inhomogeneity. Whether EIT may be useful to monitor SBT remains to be determined. No significant changes in lung ventilation, aeration or homogeneity related to extubation outcome occurred up to 30 min after extubation.

*Trial registration* Retrospectively registered on clinicaltrials.gov (Identifier: NCT03894332; release date 27th March 2019).

**Electronic supplementary material:**

The online version of this article (10.1186/s13613-019-0565-0) contains supplementary material, which is available to authorized users.

## Background

Weaning is the whole process that leads patients to the discontinuation of mechanical ventilation and the removal of the endotracheal tube [1]. Weaning should be considered as early as possible to reduce the time spent on invasive mechanical ventilation (iMV), which is associated with morbidity and mortality [2, 3]. To assess whether patients are eligible for extubation, a spontaneous breathing trial (SBT) is often performed. The outcome of SBT is evaluated by clinical assessment and acquisition of objective parameters, such as the arterial blood gases (ABGs) and the respiratory rate (RR) to tidal volume (Vt) ratio (RR/Vt) [1]. In the case of SBT success, the patient can be extubated. However, post-extubation respiratory failure may occur, in particular in patients at increased risk [1], who may benefit from prophylactic non-invasive ventilation (NIV) application immediately after extubation [4–9].

Electrical impedance tomography (EIT) is a non-invasive imaging technique that provides instantaneous monitoring of variations in overall lung volume and regional distribution of ventilation, as determined by variations over time in intrathoracic impedance, which is increased by air and reduced by fluids and cells. EIT allows for determining changes in end-expiratory lung impedance (EELI), a surrogate estimate of end-expiratory lung volume, assessing global and regional distribution of Vt, and obtaining indexes of the spatial distribution of ventilation, such as the global inhomogeneity index [10].

Because SBT failure and extubation failure are both major clinical problems, they have been repeatedly investigated, but the underlying mechanisms are not fully elucidated yet [1]. We reasoned that EIT could help comprehend the pathophysiology of SBT failure and extubation failure by providing additional insights. We, therefore, designed this observational study on patients at high risk of extubation failure, aimed at assessing variations in EELI (∆EELI) and Vt (∆Vt%), and the inhomogeneity index during SBT and after extubation.

## Methods

The study was conducted from June 2015 to June 2016 in the intensive care units (ICUs) of three Italian hospitals (“Sant’Andrea” Hospital in Vercelli, “ARNAS Garibaldi” Hospital in Catania and “Infermi” Hospital in Rimini), in accordance with the principles outlined in the Declaration of Helsinki, after approval by the local Ethics Committees (Vercelli, protocol no AslVC.Rian.14.02, approved on 11/09/2014; Catania, protocol no 479/CE, approved on 30/06/2015; Rimini, protocol no 1313, approved on 18/03/2015). Written informed consent was obtained from all patients according to national regulations.

### Patients

We included any ICU patient ≥ 18 years who (1) had received iMV for at least 48 h through an orotracheal tube, (2) was ready for the first SBT attempt [1] and (3) met at least one criterion for increased risk of extubation failure, as outlined in the first column of Additional file 1: Table S1 [4–9, 11]. Patients were considered to be ready for SBT if they satisfied all of the criteria reported in the second column of Additional file 1: Table S1 [1, 4, 12]. Patients were excluded if they met one or more of the following criteria: (1) life-threatening heart arrhythmias or cardiac ischaemia, (2) pneumothorax, (3) pulmonary emphysema, (4) recent (1 week) thoracic surgery, (5) presence of chest burns, (6) pregnancy, (7) inclusion in other research protocols, (8) refusal of consent.

Inclusion and exclusion criteria were assessed daily in all patients.

### Weaning protocol

Once able to trigger the ventilator, patients were ventilated with pressure support ventilation (PSV). Inspired fraction of oxygen (FiO_2_) and positive end-expiratory pressure (PEEP) were set to maintain peripheral oxygen saturation (SpO_2_) between 92 and 96%, while the inspiratory pressure support was titrated to generate a Vt of 6–8 ml/kg of ideal body weight with an active inspiration [13]. The SBT was conducted by setting the ventilator with 2 cm H_2_O in continuous positive airway pressure (CPAP) mode, with no inspiratory support for 30 min [12, 14]. SBT failure was defined by the presence of at least one of the criteria listed in the third column of Additional file 1: Table S1 [1, 15]. Patients who passed the SBT were immediately extubated and allowed to breathe spontaneously with a Venturi mask, while those who failed SBT were switched back to PSV with the same settings applied prior to SBT.

Predefined criteria for protocol interruption were the following: (1) haemodynamic instability (i.e., need for continuous infusion of dopamine or dobutamine > 5 mcg/kg/min, norepinephrine > 0.1 mcg/kg/min, or vasopressin to maintain mean arterial blood pressure > 60 mmHg), (2) life-threatening arrhythmias or electrocardiographic signs of ischemia, or (3) worsening of ABGs (i.e., onset of respiratory acidosis, as defined by arterial partial pressure of carbon dioxide (PaCO_2_) > 50 mmHg and pH < 7.30, or hypoxemia, as defined by arterial partial pressure of oxygen (PaO_2_) < 60 mmHg with a FiO_2_ ≥ 50%).

### Data acquisition and analysis

After enrolment, a silicon 16-electrode EIT belt of proper size was placed around the patient’s chest between the 4th and 6th intercostal spaces and connected to the EIT device (PulmoVista 500; Draeger Medical GmbH, Lübeck, Germany) [16, 17]. Signal quality was determined through dedicated built-in software. The position of EIT belt was, therefore, marked on the skin with a dermographic pencil to avoid its displacement during the study period. PSV was applied with a ventilator (Infinity V500; Draeger Medical GmbH, Lübeck, Germany) connected to the EIT device through a RS232 interface.

We acquired 5-min EIT data records at baseline (during PSV), during the first (SBT_0) and the last (SBT_30) 5 min of SBT, and when the patient was extubated during spontaneous breathing immediately (SB_0) and 30 min (SB_30) after extubation.

EIT and ventilator (i.e., airway pressure, flow and volume) data were recorded at a sample of 20 Hz, downloaded and analysed off-line on a personal computer using dedicated software (EITdiag, Draeger Medical GmbH, Lübeck, Germany). Impedance changes were calibrated against the data acquired from the ventilator by the EIT device as previously described [18]. The last 3 min of each record were analysed.

From the EIT recordings, we measured Vt, respiratory rate (RR), air distribution within the lungs, and end-expiratory lung impedance (EELI), i.e., a surrogate of the end-expiratory lung volume [19]. From EIT, we also calculated (1) Vt changes from baseline, expressed as a percentage (∆Vt%), as assessed by the EIT, (2) EELI variation (∆EELI) from baseline, expressed in ml, (3) the inhomogeneity index, which describes uneven air distribution within the lung [20], and (4) the RR/Vt at baseline, SBT_0, SBT_30, SB_0 and SB_30 [15], as derived from EIT measurements.

The inhomogeneity index was computed as follows:

$${\text{GI = }}\frac{{\sum\nolimits_{{{\text{x,y}}\,{\text{lung}}}} {\left[ {{\text{DI}}_{\text{xy}} - {\text{Median}}\left( {{\text{DI}}_{\text{lung}} } \right)} \right]} }}{{\sum\nolimits_{{{\text{x,y}}\,{\text{lung}}}} {{\text{DI}}_{\text{xy}} } }}$$

where DI is the differential impedance, DI_xy_ refers to the DI of pixels in a defined lung region and DI_lung_ represents the DI of the whole lung [20].

ABGs were obtained at baseline, SBT_30 and SB_30.

Extubation failure was defined by the occurrence of post-extubation respiratory failure, as characterized by one or more of the criteria outlined in the fourth column of Additional file 1: Table S1, in the 48 h following extubation [1, 21]. Patients who failed extubation underwent ‘‘rescue’’ CPAP by means of a high-flow (> 45 l/min) generator (Flow-Meter, StarMed, Mirandola, Modena, Italy) in the case of normocapnic hypoxemia (i.e., PaO_2_/FiO_2_ < 200 mmHg), or NIV, as delivered through NIV modality, in the case of respiratory acidosis (pH < 7.35 and PaCO_2_ > 50 mmHg). Patients were re-intubated and iMV reinstituted if one or more of the criteria displayed in the fifth column of Additional file 1: Table S1 occurred [21].

### Statistical analysis

Due to the lack of published data at the time of the study design, no power analysis could be performed. Therefore, we arbitrarily planned to enrol a sample of 80 consecutive patients. Because of the lack of normal distribution of data, as assessed by the Shapiro–Wilk test, the data were expressed as the median [25th–75th interquartile range]. Data were compared between subgroups of patients (i.e., SBT success versus failure; extubation success versus failure) using the Mann–Whitney *U*-test. Thresholds for statistical significance were adjusted with Bonferroni correction for multiple comparisons. Categorical data were compared by Fisher’s exact test; for this comparison, a two-sided *p* < 0.05 was considered significant.

Receiving operating curves (ROC) were obtained for RR/Vt and PaO_2_/FiO_2_ and EIT data. Cut-off values were obtained calculating the Youden index. The area under the curve (AUC), sensitivity, specificity, positive (LR +) and negative (LR −) likelihood ratios were also calculated.

## Results

**Fig. 1:**
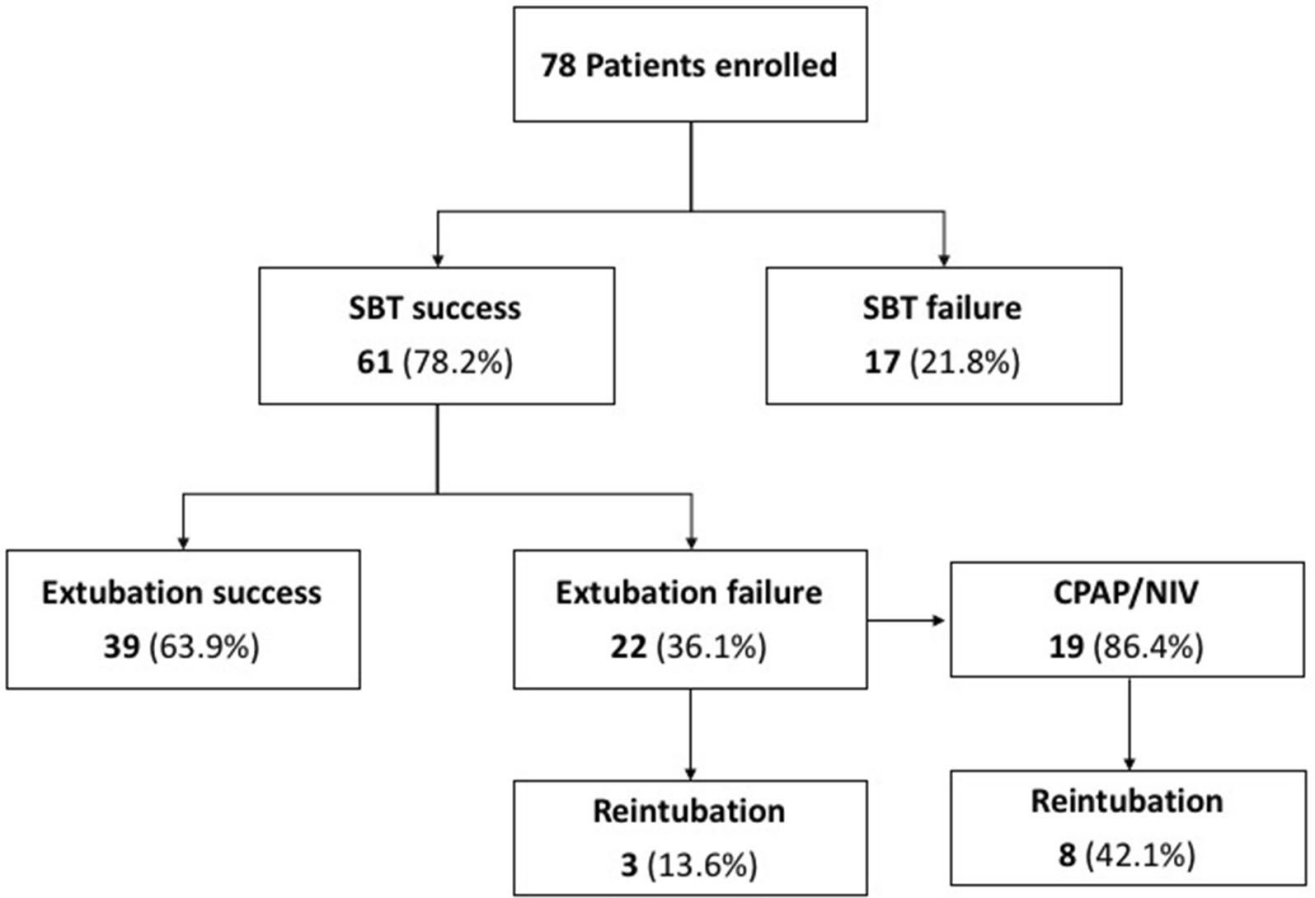
Patients’ flow throughout the study protocol. Of the overall population of 78 patients who underwent data analysis, 61 patients (78.2%) successfully passed the SBT (i.e., SBT success), whereas 17 (21.8%) did not (i.e., SBT failure). Of the 61 successfully extubated patients, 22 patients (34.9%) met one or more criteria for post-extubation respiratory failure. Among these 22 patients who experienced extubation failure, three individuals (14.9%) required immediate reintubation, while in 19 patients (86.4%) CPAP/NIV was attempted as rescue therapy. Of these 19 patients, 8 (42.1%) failed and were reintubated. Not reported in the figure is that none of the reintubated patients required tracheostomy, and only one patient died 10 days after reintubation because of septic complications

We enrolled 80 consecutive patients, as planned. Two patients, erroneously enrolled with no risk criteria for post-extubation respiratory failure, were excluded from the data analysis. The patients’ flow of study enrolment is shown in Additional file 1: Figure S1, while the protocol flow is depicted in Fig. 1. None of the patients interrupted the study protocol. Table 1 shows the patients’ characteristics at ICU admission and immediately before SBT, and the reasons for ICU admission and risk factors for post-extubation respiratory failure, for the overall population and separately for the SBT and extubation success and failure subgroups.

**Table 1:** Patient’s characteristics at ICU admission, risk factors for post-extubation respiratory failure, the reason for ICU admission and characteristics before SBT performance in the overall population and subgroups

	Overall	SBT success (*n* = 61)	SBT failure (*n* = 17)	*p* value	Extubation success (*n* = 39)	Extubation failure (*n* = 22)	*p* value
Characteristics at ICU admission							
Weight (kg)	75 [65; 83]	75 [65; 81]	75 [68; 95]	*0.458*	70 [62; 80]	80 [70; 86]	*0.128*
Height (cm)	170 [165; 173]	170 [165; 175]	170 [163; 170]	*0.286*	170 [165; 175]	170 [160; 172]	*0.203*
SAPS-II	52 [37; 67]	50 [34; 66]	59 [41; 68]	*0.188*	44 [31; 55]	63 [48; 83]	*< 0.001*
SOFA	6 [4; 9]	6 [4; 9]	4 [3; 7]	*0.049*	6 [4; 9]	7 [4; 10]	*0.790*
Risk factors for post-extubation respiratory failure							
PaCO_2_ > 45 mmHg at SBT *n* (%)	30 (38)	22 (36)	8 (47)	*0.416*	12 (31)	10 (45)	*0.279*
Chronic respiratory disease *n* (%)	17 (22)	12 (20)	5 (29)	*0.510*	7 (18)	5 (23)	*0.741*
Chronic heart failure *n* (%)	17 (22)	12 (20)	5 (29)	*0.510*	9 (23)	3 (14)	*0.510*
Upper airway stridor *n* (%)	2 (3)	2 (3)	0 (0)	*> 0.999*	2 (5)	0 (0)	*0.531*
Age ≥ 65 years *n* (%)	48 (62)	38 (62)	10 (59)	*0.786*	24 (62)	14 (64)	*> 0.999*
Cardiac failure as reason of iMV *n* (%)	10 (13)	8 (13)	2 (12)	*> 0.999*	6 (15)	2 (9)	*0.699*
APACHE-II score > 12 at extubation *n* (%)	29 (37)	24 (39)	5 (29)	*0.575*	14 (36)	10 (45)	*0.587*
ARF requiring > 72 h of iMV *n* (%)	44 (56)	30 (49)	14 (82)	*0.025*	19 (49)	11 (48)	*> 0.999*
BMI > 35 kg/m^2^ *n* (%)	11 (14)	5 (8)	6 (35)	*0.011*	3 (8)	2 (9)	*> 0.999*
Neuromuscular disease *n* (%)	9 (12)	4 (7)	5 (29)	*0.020*	3 (8)	1 (5)	*> 0.999*
Reason for ICU admission							
Acute heart failure *n* (%)	10 (13)	6 (10)	4 (23)	*0.212*	0 (0)	6 (27)	*0.013*
Acute on Chronic respiratory failure *n* (%)	17 (22)	12 (20)	5 (29)	*0.507*	7 (18)	5 (23)	*0.742*
Sepsis/septic shock *n* (%)	21 (27)	20 (33)	1 (6)	*0.031*	14 (36)	6 (27)	*0.577*
Trauma *n* (%)	10 (13)	7 (11)	3 (18)	*0.682*	7 (18)	0 (0)	*0.042*
Pneumonia *n* (%)	20	16 (26)	4 (24)	*> 0.999*	11 (28)	5 (23)	*0.766*
Characteristics before the SBT							
PEEP (cmH_2_O)	5.0 [5.0; 5.1]	5.0 [5.0; 5.1]	5.0 [5.0; 5.0]	*0.168*	5.0 [5.0; 5.1]	5.0 [5.0; 5.1]	*0.953*
Inspiratory support (cmH_2_O)	10 [8; 11]	10 [8; 10]	10 [9; 11]	*0.531*	9 [8; 11]	10 [10; 10]	*0.118*
pH	7.44 [7.41; 7.47]	7.44 [7.40; 7.48]	7.46 [7.42; 7.48]	*0.546*	7.45 [7.41; 7.48]	7.42 [7.40; 7.45]	*0.397*
PaCO_2_ (mmHg)	39.3 [36.3; 45.0]	39.3 [36.0; 45.0]	39.5 [38.2; 44.4]	*0.392*	38.5 [34.0; 44.3]	39.3 [38.0; 47.3]	*0.176*
PaO_2_/FiO_2_ (mmHg)	268 [230; 310]	274 [241; 306]	248 [205; 324]	*0.347*	274 [243; 303]	270 [226; 313]	*0.932*

SBT: Spontaneous breathing trial; ICU: intensive care unit; SAPS-II: simplified acute physiology score II; SOFA: sequential organ failure assessment; PaCO_2_: arterial partial pressure of carbon dioxide; iMV: invasive mechanical ventilation; APACHE-II: acute physiology and chronic health evaluation II; ARF: acute respiratory failure; BMI: body mass index; PEEP: positive-end expiratory pressure; PaO_2_/FiO_2_: ratio between arterial partial pressure and inspired fraction of oxygen

### SBT success and failure

Seventeen patients (21.8%) failed SBT and were returned to iMV. At baseline, PEEP values were similar between groups (5.0 [4.9; 5.1] cm H_2_O and 5.0 [5.0; 5.0] cm H_2_O for SBT success and failure, respectively; *p* = 0.168). Table 2 reports ABGs and RR/Vt separately for SBT success and failure. ABGs were not significantly different between groups at both baseline and SBT_30. RR/Vt was also not different between the groups at baseline but was significantly greater at SBT_0 and SBT_30 in SBT failures compared to SBT success (*p* < 0.001 for both).

**Table 2:** Arterial blood gases and the rapid shallow breathing index (RR/Vt) in patients with SBT success and failure

	SBT success (*n* = 61)			SBT failure (*n* = 17)			
	Baseline	SBT_0	SBT_30	Baseline	SBT_0	SBT_30	SBT success vs. failure
pH	7.44 [7.40; 7.48]	//	7.43 [7.39; 7.47]	7.46 [7.42; 7.48]	//	7.41 [7.38; 7.48]	*p = 0.546* ^*a*^ *p = 0.762* ^*c*^
PaCO_2_ (mmHg)	39.3 [36.0; 45.0]	//	40.0 [35.2; 46.0]	39.5 [38.2; 44.4]	//	41.0 [38.3; 53.5]	*p = 0.392* ^*a*^ *p = 0.194* ^*c*^
PaO_2_/FiO_2_ (mmHg)	274 [241; 306]	//	253 [221; 291]	248 [205; 324]	//	188 [156; 237]	*p = 0.347* ^*a*^ *p = 0.034* ^*c*^
RR/Vt (breaths/min/L)	35 [25; 57]	57 [38; 84]	60 [33; 92]	46 [26; 64]	102 [70; 135]	103 [75; 127]	*p = 0.389* ^*a*^ *p < 0.001* ^*b*^ *p < 0.001* ^*c*^

SBT, Spontaneous Breathing Trial; SBT_0, first 5 min after the beginning of the SBT; SBT_30, last 5 min of the SBT; PaCO_2_, arterial partial pressure of carbon dioxide; PaO_2_/FiO_2_, arterial partial pressure to inspired fraction of oxygen ratio; RR/Vt, respiratory rate to tidal volume ration

//Data not present

^a^Comparison between groups within baseline

^b^Comparison between groups within SBT_0

^c^Comparison between groups within SBT_30. According to Bonferroni correction, the threshold for statistical significance is *p* < 0.025 for pH, PaCO_2_ and PaO_2_/FiO_2_, while *p* < 0.017 for RR/Vt

**Fig. 2:**
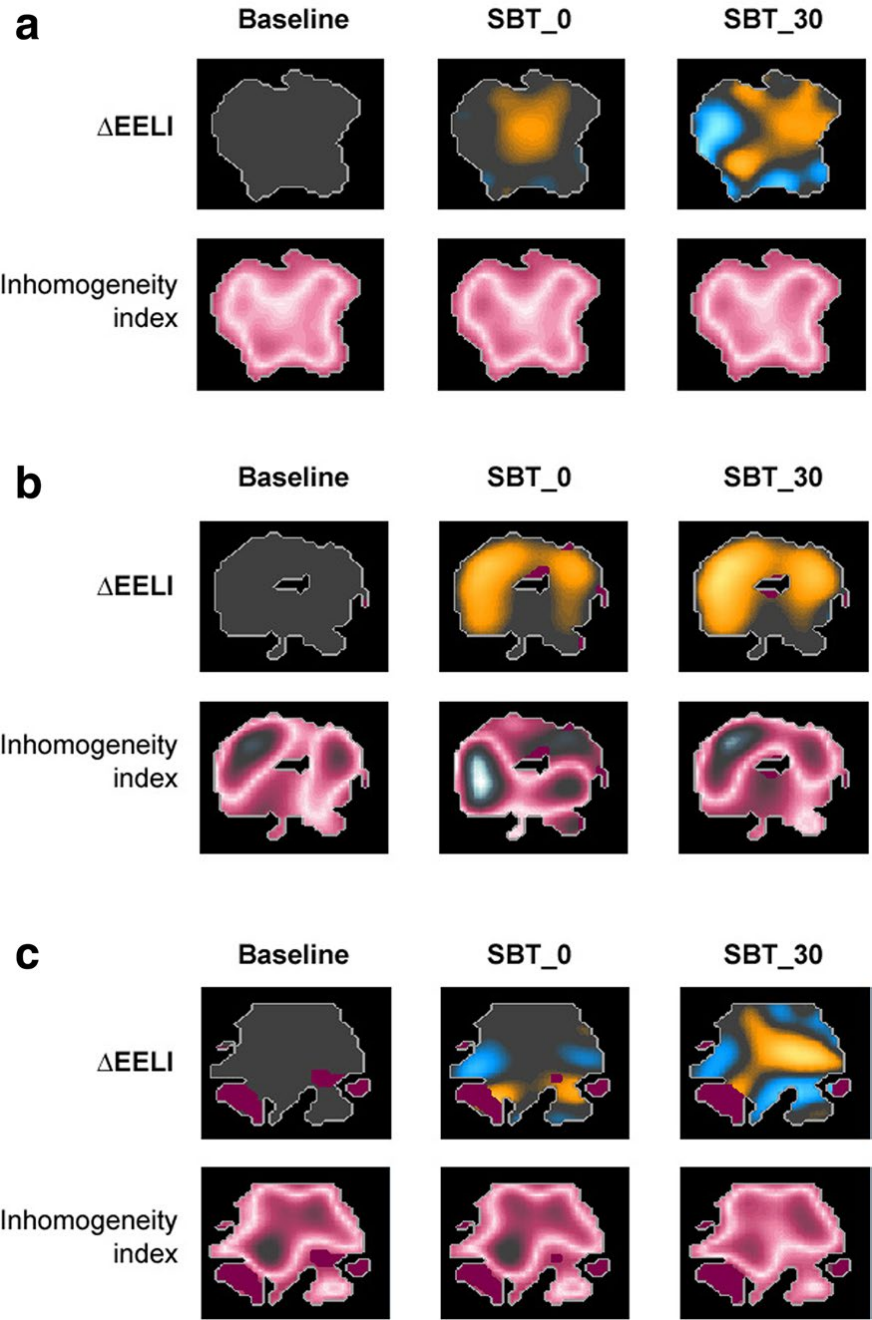
EIT images from three representative patients. Examples of EIT images from three representative patients, two succeeding (**a**, **c**) and one failing (**b**) the SBT. ∆EELI and the inhomogeneity index are shown at baseline, SBT_0 and SBT_30. For ∆EELI, the yellow and blue colour codes indicate EELI loss and increase, respectively. A pink scale code depicts inhomogeneity of gas distribution within the lungs. **a** Characterized by a relatively small ∆EELI, as referenced to baseline, at both SBT_0 (− 181 ml) and SBT_30 (− 178 ml). The inhomogeneity index is stable, ranging between 35 and 40 in all three trials. In **b**, conversely, ∆EELI is much greater (− 752 ml at SBT_0 and − 824 ml at SBT_30), indicating a remarkable loss of end-expiratory lung volume at both time points. The pink colour is quite scattered, indicating elevated values of inhomogeneity (54, 72 and 69, at baseline, SBT_0 and SBT_30, respectively). In **c**, ∆EELI does not significantly change (− 110 ml at SBT_0 and − 117 at SBT_30), while the inhomogeneity index is increased (68, 70 and 71 at baseline, SBT_0 and SBT_30, respectively). Notably, in contrast to the two previous examples, this patient was affected by chronic obstructive pulmonary disease

Examples of EIT images from three representative patients, two succeeding (panels A and C) and one failing (panel B) SBT, are depicted in Fig. 2; ∆EELI and the inhomogeneity index are shown at baseline, SBT_0 and SBT_30 (see figure legend for detailed information). Table 3 displays the values of ∆Vt%, ∆EELI and inhomogeneity index in SBT success and failure, respectively. The ∆Vt% was no different at baseline (*p* > 0.999), SBT_0 (*p* = 0.079) and SBT_30 (*p* = 0.054). Compared to SBT successes, SBT failures showed a greater reduction in ∆EELI at SBT_0 (*p* < 0.001) and SBT_30 (*p* = 0.001), and a higher inhomogeneity index at baseline (*p* = 0.002), SBT_0 (*p* = 0.003) and SBT_30 (*p* = 0.005).

**Table 3:** EIT data in patients with SBT success and failure

	SBT success (*n* = 61)			SBT failure (*n* = 17)			Success vs. failure
	Baseline	SBT_0	SBT_30	Baseline	SBT_0	SBT_30	
∆Vt%	0 [0; 0]	− 16.4 [− 37.0; − 2.5]	− 12.9 [− 35.0; 5.0]	0 [0; 0]	− 27.6 [− 51.0; − 16.0]	− 22.9 [− 50.6; − 13.3]	*p > 0.999* ^*a*^ *p = 0.079* ^*b*^ *p = 0.054* ^*c*^
∆EELI (ml)	0 [0; 0]	− 117 [− 240; 21]	− 103 [− 292; 62]	0 [0; 0]	− 456 [− 934; − 162]	− 333 [− 1375; − 157]	*p > 0.999* ^*a*^ *p < 0.001* ^*b*^ *p = 0.001* ^*c*^
Inhomogeneity index	51 [44; 61]	56 [48; 71]	57 [46; 70]	65 [54; 87]	89 [61; 105]	90 [62; 101]	*p = 0.002* ^*a*^ *p = 0.003* ^*b*^ *p = 0.005* ^*c*^

SBT: Spontaneous Breathing Trial; SBT_0: first 5 min after the beginning of the SBT; SBT_30: last 5 min of the SBT; ∆Vt%: change from baseline of the tidal volume in percentage; ∆EELI: change from baseline of the end-expiratory lung impedance

^a^Comparison between groups within baseline

^b^Comparison between groups within SBT_0

^c^Comparison between groups within SBT_30. According to Bonferroni correction, the threshold for statistical significance is *p* < 0.017

The AUC, sensitivity, specificity, LR + and LR − of ∆Vt%, ∆EELI, inhomogeneity index, RR/Vt and PaO_2_/FiO_2_ for SBT failure prediction are shown in Additional file 1: Table S2.

### Extubation success and failure

Twenty-two of 61 (36.1%) patients extubated after SBT success met criteria for extubation failure: 15 for ABG worsening and respiratory distress and 7 for non-respiratory reasons (5 for neurological deterioration, one for inability to remove secretions and one for haemodynamic instability). Eleven patients were successfully treated by CPAP or NIV, while 11 required endotracheal intubation, 3 immediately and 8 after an unsuccessful attempt of CPAP or NIV.

As shown in Additional file 1: Table S3, there were no differences between extubation success and failure in pH (*p* = 0.397 at baseline; *p* = 0.453 at SBT_30; *p* = 0.179 at SB_30), PaCO_2_ (*p* = 0.176 at baseline; *p* = 0.467 at SBT_30; *p* = 0.757 at SB_30) and PaO_2_/FiO_2_ (*p* = 0.932 at baseline; *p* = 0.636 at SBT_30; *p* = 0.139 at SB_30). RR/VT was also not different among trials (*p* = 0.712 at baseline; *p* = 0.704 at SBT_0; *p* = 0.597 at SBT_30; *p* = 0.059 at SB_0; *p* = 0.185 at SB_30).

Table 4 reports the ∆Vt%, ∆EELI and inhomogeneity index for extubation success and failure. No differences were observed for any of these variables between extubation success and failure.

**Table 4:** EIT data in patients with extubation success and failure

	Baseline	SBT_0	SBT_30	SB_0	SB_30
∆Vt%					
Extubation success (*n* = 39)	0 [0; 0]	− 12 [− 31; 0]	− 9 [− 29; 7]	− 3 [− 16; 18]	− 12 [− 34; 19]
Extubation failure (*n* = 22)	0 [0; 0]	− 25 [− 42; − 10]	− 29 [− 45; − 11]	− 21 [− 39; 1]	− 22 [− 46; − 11]
Extubation success vs. failure	*p > 0.999*	*p = 0.117*	*p = 0.024*	*p = 0.014*	*p = 0.061*
∆EELI (ml)					
Extubation success (*n* = 39)	0 [0; 0]	− 125 [− 237; 23]	− 78 [− 250; 115]	− 187 [− 488; 82]	− 60 [− 371; 256]
Extubation failure (*n* = 22)	0 [0; 0]	− 112 [− 316; 17]	− 194 [− 335; − 59]	− 236 [− 438; 139]	− 290 [− 537; 152]
Extubation success vs. failure	*p > 0.999*	*p = 0.671*	*p = 0.253*	*p > 0.999*	*p = 0.132*
Inhomogeneity index					
Extubation success (*n* = 39)	46 [42; 63]	53 [44; 68]	56 [44; 70]	52 [45; 66]	53 [45; 69]
Extubation failure (*n* = 22)	53 [48; 60]	65 [55; 80]	62 [54; 71]	64 [50; 79]	66 [55; 74]
Extubation success vs. failure	*p = 0.166*	*p = 0.025*	*p = 0.132*	*p = 0.049*	*p = 0.029*

SBT: Spontaneous Breathing Trial; SBT_0: first 5 min after the beginning of the SBT; SBT_30: last 5 min of the SBT; SB_0: first 5 min of the spontaneous breathing; SB_30: last 5 min of the spontaneous breathing; ∆Vt%: change from baseline of the tidal volume in percentage; ∆EELI: change from baseline of the end-expiratory lung impedance

According to Bonferroni correction, the threshold for statistical significance is *p* < 0.017

The AUC, sensitivity, specificity, LR + and LR − of ∆Vt%, ∆EELI, inhomogeneity index, RR/Vt and PaO_2_/FiO_2_ for extubation failure prediction are shown in Additional file 1: Table S4. In addition, as reported in Additional file 1: Tables S5 and S6, we found no difference in any EIT parameters between patients succeeding and failing NIV and between patients with post-extubation respiratory failure due to respiratory and non-respiratory causes.

## Discussion

In this physiological study aimed at describing, in a general population of critically ill patients at the first SBT attempt, changes in lung aeration, ventilation distribution and inhomogeneity occurring during the weaning process, we found the following: (1) compared to SBT success, SBT failure is characterized by more lung de-recruitment, as suggested by the higher loss in ∆EELI and by the concomitant PaO_2_/FiO_2_ worsening, and greater lung inhomogeneity, as indicated by higher inhomogeneity index value; and (2) no EIT variables were significantly different between patients succeeding and failing extubation.

All patients considered ready-to-wean were considered eligible for inclusion in this study, and we did not focus only on patients with prolonged weaning, who represent only approximately 15% of the overall ICU population [1]. We found an SBT failure rate of 21% which was not different from those previously reported [22–24], while in keeping with Ferrer et al. [6], the rate of extubation failure in our population at high risk of post-extubation respiratory failure amounted to 35%. Finally, consistent with recently published data in patients recovering from hypoxemic acute respiratory failure [25], 50% of all patients experiencing post-extubation respiratory failure required reintubation, 42% of whom after failing CPAP or NIV.

The application of EIT during weaning has been recently reported only in small populations of patients characterized by prolonged weaning or prolonged mechanical ventilation [26–28]. These studies report that, compared to patients undergoing a successful SBT, patients experiencing SBT failure are characterized by greater loss of EELI at the end of the SBT. While confirming this finding in a less selected patient population, we find that the loss of EELI occurs after only 5 min.

In the present study, the inhomogeneity index appears to be greater in SBT failure, as opposed to SBT success, which is also consistent with previously published data. In patients who had received prolonged iMV, Zhao et al. [27] also found that weaning was more likely to be successful when the ventilation distribution was more homogeneous at decreasing support levels. In 31 patients with prolonged weaning, Bickenbach et al. [26] found the inhomogeneity index to be greater in SBT failures, as opposed to successes. In our study, the inhomogeneity index was also significantly higher at baseline, while RR/Vt showed significant differences only in the course of SBT. In addition, after 5 min of SBT, we observed a remarkable increase in the inhomogeneity index, without important changes at SBT completion.

We also investigated potential variations in EIT parameters related to the outcome of extubation but found no differences in any of the EIT parameters considered between patients who succeeded and failed extubation. This finding may suggest, on the one hand, that the process leading to failure takes more than the 30-min period we observed in the present study, while on the other hand, that factors other than alterations of ventilation and homogeneity intervene in the pathophysiology of post-extubation respiratory failure. Indeed, in 7 patients (32%), the primary causes of extubation were either unmanageable secretions or haemodynamic instability or neurologic deterioration. In addition, we failed to observe differences in any EIT parameters between patients who succeeded and failed NIV as rescue treatment of overt post-extubation respiratory failure.

Our study has some points of strength. First, in contrast to previous investigations [26–28], this is a multicentre study. Second, to reduce the risk of bias, the data analysis was performed in only one centre by a single investigator unaware of SBT and extubation outcomes. Third, we were extremely careful in maintaining the position of the EIT belt throughout the study period by marking the skin with a dermographic pencil, to prevent the risk of belt displacement, which is a well-known source of bias [10].

Our study also includes some limitations. We did not calculate the sample size because of the lack of published data at the time this study was designed and initiated. We did not register the trial prior to patient enrolment because the registration was not mandatory for observational studies when the study was initiated. We did not record the amount of fluids administered in the course of the study protocol. Fluid overload alters the measurement of EELI, mimicking a reduction in end-expiratory lung volume. One study in pigs showed that a rapid infusion (33 ml/kg/h) of fluids caused a significant reduction in overall relative impedance [29]. In another animal study, after a rapid (approximately 6 min) infusion of 500 ml of crystalloids the authors observed a decrease in EELI, corresponding to a mean virtual reduction in end-expiratory lung volume of 227 ml [30]. Conversely, a sudden increase in urine output up to 1200 ml consequent to a bolus of diuretics [31] or a loss of volume during haemodialysis [32] may determine EELI to increase. It should be noted, however, that a stable cardiovascular condition was a prerequisite for SBT eligibility in our study, which makes it highly unlikely that our patients received rapid liquid infusion or removal. In addition, none of the patients enrolled required haemodialysis or continuous veno-venous haemofiltration.

## Conclusions

SBT failure is overall characterized early in the course of SBT by a greater reduction in EELI reflecting a fall in end-expiratory lung volume and more inhomogeneity of ventilation distribution, as opposed to patients succeeding SBT, but our data do not identify clear-cut thresholds. Whether EIT may be a useful adjunctive tool to monitor SBT remains to be determined by specifically designed clinical studies. No significant changes in lung aeration, ventilation distribution or homogeneity related to extubation outcome occurs up to 30 min after extubation.

## **Additional file**

**Additional file 1: Table S1.** Criteria considered in the study protocol. Criteria for increased risk for post-extubation respiratory failure, SBT eligibility, SBT failure, post-extubation respiratory failure and reintubation are presented from left to right. **Table S2.** Receiving Operating Curves of ∆Vt%, ∆EELI, inhomogeneity index, RR/Vt and PaO_2_/FiO_2_ for SBT failure prediction. The Youden index, area under the curve (AUC), sensibility, specificity, positive (LR +) and negative (LR −) likelihood ratios are presented for EIT data, RR/Vt and PaO_2_/FiO_2_ for SBT failure prediction. **Table S3.** ABGs and RR/Vt in patients with extubation success and failure. Data are separately presented for patients succeeding and failing extubation. **Table S4.** Receiving Operating Curves of ∆Vt%, ∆EELI, inhomogeneity index, RR/Vt and PaO_2_/FiO_2_ for extubation failure prediction. The Youden index, area under the curve (AUC), sensibility, specificity, positive (LR +) and negative (LR −) likelihood ratios are presented for EIT data, RR/Vt and PaO_2_/FiO_2_ for extubation failure prediction. **Table S5.** EIT data in patients with “rescue” NIV success and failure. EIT parameters are separately presented for patients succeeding and failing rescue CPAP/NIV. **Table S6.** EIT data in patients with respiratory and non-respiratory reasons of extubation failure. EIT parameters are separately presented for patients with extubation failure secondary to respiratory and non-respiratory causes. **Figure S1.** Flow diagram of screened and enrolled patients. The flow of patients screened for the study (*n* = 1555), assessed for eligibility (*n* = 145), excluded (with the reason of exclusion) (*n* = 65), enrolled in the study (*n* = 80), and analyzed (*n* = 78) is shown.

## Data Availability

The full protocol, datasets used and analysed during the current study are available on reasonable request at longhini.federico@gmail.com.

## Abbreviations

ABGs: arterial blood gases
CPAP: continuous positive airway pressure
∆EELI: changes in milliliters from baseline of the end-expiratory lung impedance
∆Vt%: variation in percentage from baseline of the tidal volume
EELI: end-expiratory lung impedance
EIT: electrical impedance tomography
ESM: electronic supplementary materials
FiO_2_: inspired fraction of oxygen
ICU: intensive care unit
iMV: invasive mechanical ventilation
NIV: non-invasive ventilation
PaCO_2_: arterial partial pressure of carbon dioxide
PaO_2_: arterial partial pressure of oxygen
PEEP: positive end-expiratory pressure
PSV: pressure support ventilation
RR: respiratory rate
SB_0: first 5 min of the spontaneous breathing
SB_30: last 5 min of the spontaneous breathing
SBT_0: first 5 min after SBT initiation
SBT_30: last 5 min of the SBT
SBT: spontaneous breathing trial
SpO_2_: peripheral oxygen saturation
Vt: tidal volume

## References

[1] Boles JM, Bion J, Connors A, Herridge M, Marsh B, Melot C (2007). Weaning from mechanical ventilation. Eur Respir J.

[2] Ely EW, Baker AM, Dunagan DP, Burke HL, Smith AC, Kelly PT (1996). Effect on the duration of mechanical ventilation of identifying patients capable of breathing spontaneously. N Engl J Med.

[3] Esteban A, Anzueto A, Frutos F, Alia I, Brochard L, Stewart TE (2002). Characteristics and outcomes in adult patients receiving mechanical ventilation: a 28-day international study. JAMA.

[4] Nava S, Gregoretti C, Fanfulla F, Squadrone E, Grassi M, Carlucci A (2005). Noninvasive ventilation to prevent respiratory failure after extubation in high-risk patients. Crit Care Med.

[5] Ferrer M, Sellares J, Valencia M, Carrillo A, Gonzalez G, Badia JR (2009). Non-invasive ventilation after extubation in hypercapnic patients with chronic respiratory disorders: randomised controlled trial. Lancet.

[6] Ferrer M, Valencia M, Nicolas JM, Bernadich O, Badia JR, Torres A (2006). Early noninvasive ventilation averts extubation failure in patients at risk: a randomized trial. Am J Respir Crit Care Med.

[7] El-Solh AA, Aquilina A, Pineda L, Dhanvantri V, Grant B, Bouquin P (2006). Noninvasive ventilation for prevention of post-extubation respiratory failure in obese patients. Eur Respir J.

[8] Vianello A, Arcaro G, Braccioni F, Gallan F, Marchi MR, Chizio S (2011). Prevention of extubation failure in high-risk patients with neuromuscular disease. J Crit Care.

[9] Ornico SR, Lobo SM, Sanches HS, Deberaldini M, Tofoli LT, Vidal AM (2013). Noninvasive ventilation immediately after extubation improves weaning outcome after acute respiratory failure: a randomized controlled trial. Crit Care.

[10] Lobo B, Hermosa C, Abella A, Gordo F (2018). Electrical impedance tomography. Ann Transl Med.

[11] Longhini F, Pan C, Xie J, Cammarota G, Bruni A, Garofalo E (2017). New setting of neurally adjusted ventilatory assist for noninvasive ventilation by facial mask: a physiologic study. Crit Care.

[12] Navalesi P, Frigerio P, Moretti MP, Sommariva M, Vesconi S, Baiardi P (2008). Rate of reintubation in mechanically ventilated neurosurgical and neurologic patients: evaluation of a systematic approach to weaning and extubation. Crit Care Med.

[13] Foti G, Cereda M, Banfi G, Pelosi P, Fumagalli R, Pesenti A (1997). End-inspiratory airway occlusion: a method to assess the pressure developed by inspiratory muscles in patients with acute lung injury undergoing pressure support. Am J Respir Crit Care Med.

[14] Vaschetto R, Frigerio P, Sommariva M, Boggero A, Rondi V, Grossi F (2015). Evaluation of a systematic approach to weaning of tracheotomized neurological patients: an early interrupted randomized controlled trial. Ann Intensive Care.

[15] Yang KL, Tobin MJ (1991). A prospective study of indexes predicting the outcome of trials of weaning from mechanical ventilation. N Engl J Med.

[16] Bikker IG, Preis C, Egal M, Bakker J, Gommers D (2011). Electrical impedance tomography measured at two thoracic levels can visualize the ventilation distribution changes at the bedside during a decremental positive end-expiratory lung pressure trial. Crit Care.

[17] Costa EL, Lima RG, Amato MB (2009). Electrical impedance tomography. Curr Opin Crit Care.

[18] Lowhagen K, Lundin S, Stenqvist O (2010). Regional intratidal gas distribution in acute lung injury and acute respiratory distress syndrome assessed by electric impedance tomography. Minerva Anestesiol.

[19] Mauri T, Eronia N, Abbruzzese C, Marcolin R, Coppadoro A, Spadaro S (2015). Effects of sigh on regional lung strain and ventilation heterogeneity in acute respiratory failure patients undergoing assisted mechanical ventilation. Crit Care Med.

[20] Zhao Z, Moller K, Steinmann D, Frerichs I, Guttmann J (2009). Evaluation of an electrical impedance tomography-based global inhomogeneity index for pulmonary ventilation distribution. Intensive Care Med.

[21] Esteban A, Frutos-Vivar F, Ferguson ND, Arabi Y, Apezteguia C, Gonzalez M (2004). Noninvasive positive-pressure ventilation for respiratory failure after extubation. N Engl J Med.

[22] Esteban A, Alia I, Gordo F, Fernandez R, Solsona JF, Vallverdu I (1997). Extubation outcome after spontaneous breathing trials with T-tube or pressure support ventilation. The Spanish Lung Failure Collaborative Group. Am J Respir Crit Care Med.

[23] Esteban A, Frutos F, Tobin MJ, Alia I, Solsona JF, Valverdu I (1995). A comparison of four methods of weaning patients from mechanical ventilation. Spanish Lung Failure Collaborative Group. N Engl J Med.

[24] Brochard L, Rauss A, Benito S, Conti G, Mancebo J, Rekik N (1994). Comparison of three methods of gradual withdrawal from ventilatory support during weaning from mechanical ventilation. Am J Respir Crit Care Med.

[25] Vaschetto R, Longhini F, Persona P, Ori C, Stefani G, Liu S (2019). Early extubation followed by immediate noninvasive ventilation vs. standard extubation in hypoxemic patients: a randomized clinical trial. Intensive Care Med.

[26] Bickenbach J, Czaplik M, Polier M, Marx G, Marx N, Dreher M (2017). Electrical impedance tomography for predicting failure of spontaneous breathing trials in patients with prolonged weaning. Crit Care.

[27] Zhao Z, Peng SY, Chang MY, Hsu YL, Frerichs I, Chang HT (2017). Spontaneous breathing trials after prolonged mechanical ventilation monitored by electrical impedance tomography: an observational study. Acta Anaesthesiol Scand.

[28] Hsu YL, Tien AJ, Chang MY, Chang HT, Moller K, Frerichs I (2017). Regional ventilation redistribution measured by electrical impedance tomography during spontaneous breathing trial with automatic tube compensation. Physiol Meas.

[29] Bodenstein M, Wang H, Boehme S, Vogt A, Kwiecien R, David M (2012). Influence of crystalloid and colloid fluid infusion and blood withdrawal on pulmonary bioimpedance in an animal model of mechanical ventilation. Physiol Meas.

[30] Sobota V, Muller M, Roubik K (2019). Intravenous administration of normal saline may be misinterpreted as a change of end-expiratory lung volume when using electrical impedance tomography. Sci Rep.

[31] Noble TJ, Harris ND, Morice AH, Milnes P, Brown BH (2000). Diuretic induced change in lung water assessed by electrical impedance tomography. Physiol Meas.

[32] Kunst PW, Vonk Noordegraaf A, Straver B, Aarts RA, Tesselaar CD, Postmus PE (1998). Influences of lung parenchyma density and thoracic fluid on ventilatory EIT measurements. Physiol Meas.

