# Supplementary material for: Electrical impedance tomography during spontaneous breathing trials and after extubation in critically ill patients at high risk for extubation failure: a multicenter observational study
Source: Ann Intensive Care. 2019 Aug 13;9:88. doi: 10.1186/s13613-019-0565-0 (PMC6692788; doi:10.1186/s13613-019-0565-0)
Supplement: Supplementary file 1 — Additional file 1: Table S1. Criteria considered in the study protocol. Criteria for increased risk for post-extubation respiratory failure, SBT eligibility, SBT failure, post-extubation respiratory failure and reintubation are presented from left to right. Table S2. Receiving Operating Curves of ∆Vt%, ∆EELI, inhomogeneity index, RR/Vt and PaO2/FiO2 for SBT failure prediction. The Youden index, area under the curve (AUC), sensibility, specificity, positive (LR +) and negative (LR −) likelihood ratios are presented for EIT data, RR/Vt and PaO2/FiO2 for SBT failure prediction. Table S3. ABGs and RR/Vt in patients with extubation success and failure. Data are separately presented for patients succeeding and failing extubation. Table S4. Receiving Operating Curves of ∆Vt%, ∆EELI, inhomogeneity index, RR/Vt and PaO2/FiO2 for extubation failure prediction. The Youden index, area under the curve (AUC), sensibility, specificity, positive (LR +) and negative (LR −) likelihood ratios are presented for EIT data, RR/Vt and PaO2/FiO2 for extubation failure prediction. Table S5. EIT data in patients with “rescue” NIV success and failure. EIT parameters are separately presented for patients succeeding and failing rescue CPAP/NIV. Table S6. EIT data in patients with respiratory and non-respiratory reasons of extubation failure. EIT parameters are separately presented for patients with extubation failure secondary to respiratory and non-respiratory causes. Figure S1. Flow diagram of screened and enrolled patients. The flow of patients screened for the study (n = 1555), assessed for eligibility (n = 145), excluded (with the reason of exclusion) (n = 65), enrolled in the study (n = 80), and analyzed (n = 78) is shown. [file 13613_2019_565_MOESM1_ESM.docx]

**ELECTRICAL IMPEDANCE TOMOGRAPHY DURING SPONTANEOUS BREATHING TRIALS AND AFTER EXTUBATION IN CRITICALLY ILL PATIENTS AT HIGH RISK FOR EXTUBATION FAILURE: A MULTICENTER OBSERVATIONAL STUDY.**

**Additional file**

Federico Longhini, MD^1^; Jessica Maugeri, MD ^2^; Cristina Andreoni, MD ^3^; Chiara Ronco, MD ^1^; Andrea Bruni, MD ^4^; Eugenio Garofalo, MD ^4^; Corrado Pelaia, MD ^4^; Camilla Cavicchi, MD ^3^; Sergio Pintaudi, MD ^2^; Paolo Navalesi, MD, PhD, FERS ^4^.

**Affiliations:**^1^Anesthesia and Intensive Care, Sant’Andrea Hospital, ASL VC, Vercelli, Italy; ^2^ Anesthesia and Intensive Care, "Garibaldi Centro" Hospital, ARNAS Garibaldi, Catania, Italy; ^3^ Anesthesia and Intensive Care, Infermi Hospital, AUSL Romagna, Rimini, Italy; ^4^Intensive Care Unit, University Hospital Mater Domini, Department of Medical and Surgical Sciences, Magna Graecia University, Catanzaro, Italy.

**Corresponding author:**

Prof. Paolo Navalesi, MD, FERS

Intensive Care Unit, University Hospital Mater Domini

Department of Medical and Surgical Sciences

Magna Graecia University

Viale Europa - Loc. Germaneto 88100, Catanzaro, Italy

E-mail: pnavalesi@unicz.it

Tel: +393355321910

**Table S1. Criteria considered in the study protocol**

| **Risk for post-extubation respiratory failure** | **SBT Eligibility** | **SBT Failure** | **Post-extubation respiratory failure** | **Reintubation** |
| --- | --- | --- | --- | --- |
| 1) PaCO_2_ >45 mmHg at SBT end  2) chronic respiratory disorders  3) chronic heart failure  4) upper airway stridor at extubation not requiring immediate reintubation  5) age ≥65 years  6) cardiac failure as reason of intubation  7) APACHE-II score >12 on extubation day  8) acute respiratory failure requiring >72 h of iMV  9) BMI >35 kg/m^2^  10) presence of a neuromuscular disease | 1) GCS ≥8  2) presence of clearly audible cough during suctioning with need for tracheal suctioning ≤2/hour  3) normal sodium blood values  4) core temperature <38.5° during the previous 8 hours  5) PaO_2_/FiO_2_ ≥200 mmHg, with PEEP ≤5 cmH_2_O and FiO_2_ ≤0.4  7) stable cardiovascular status (*i.e.,* HR ≤140 beats/min, sBP between 90 and 160 mmHg without need for vasopressin, epinephrine or norepinephrine infusion, or with dopamine or dobutamine infusion ≤5 mcg/kg/min)  8) cuff leak volume >110 mL | 1) presence of mental status alteration, agitation, anxiety or loss of ≥ 2 points of GCS  2) RR/Vt ≥105 breaths/min/L  3) PaO_2_ ≤60 mmHg on FiO_2_ ≥0.5 and/or pH <7.32 or a decrease in pH ≥0.07 units at the end of the SBT  4) sBP <90 mmHg or ≥180 mmHg or increased by ≥20%  5) HR >140 beats/min or increased by 20%  6) onset of major heart arrhythmias, or electrocardiographic signs of cardiac ischemia  7) RR ≥35 breaths/min or increased by ≥50%  8) increased effort, respiratory distress (as indicated by diaphoresis, accessory respiratory muscles recruitment, facial signs of distress and/or paradoxical breath) | 1) RR >25 breaths/min for 2 hours  2) HR >140 beats/min or sustained increase or decrease >20%  3) clinical signs of respiratory muscle failure or increased work of breathing  4) SpO_2_ <90% or PaO_2_ <80 mmHg on FiO_2_ ≥50%  5) PaCO_2_ >45 mmHg or increase ≥20% from pre-extubation with pH <7.33 | 1) need for emergency intubation  2) lack of improvement in gas exchange or in signs of respiratory-muscle failure with CPAP/NIV  3) mental status alterations, rendering the patient unable to tolerate CPAP/NIV  4) decrease in the SpO_2_ <85% on FiO_2_ ≥50%  5) need for continuous infusion of dopamine or dobutamine >5 mcg/kg/min, norepinehrine > 0.1 mcg/kg/min or vasopressin to maintain mean arterial blood pressure > 60 mmHg  6) copious and unmanageable secretions |

SBT, spontaneous breathing trial; PaCO_2_, arterial partial pressure of carbon dioxide; APACHE-II, Acute Physiology and Chronic Health Evaluation II; iMV, invasive Mechanical Ventilation; BMI, Body Mass Index; GCS, Glasgow Coma Scale; PaO_2_/FiO_2_, oxygen arterial tension to inspired fraction ratio; PEEP, Positive End-Expiratory Pressure; FiO_2_ oxygen inspired fraction; HR, heart rate; sBP, systolic blood pressure; RR, respiratory rate; Vt, tidal volume; PaO_2_, oxygen arterial tension; SpO_2_, peripheral oxygen saturation; CPAP, Continuous Positive Airway Pressure; NIV, Non Invasive Ventilation.

**Table S2. Receiving Operating Curves of ΔVt%, ΔEELI, inhomogeneity index, RR/Vt and PaO_2_/FiO_2_ for SBT failure prediction.**

| **Parameter** | **Youden index** | **AUC (95% CI)** | **P value** | **Sensitivity (95% CI)** | **Specificity (95% CI)** | **LR+ (95% CI)** | **LR- (95% CI)** |
| --- | --- | --- | --- | --- | --- | --- | --- |
| **ΔVt% (%)** | | | | | | | |
| *SBT_0* | ≤15.9 | 0.65 (0.53-0.75) | 0.056 | 82.4 (56.6-96.2) | 50.8 (37.9-63.6) | 1.7 (1.2-2.3) | 0.4 (0.1-1.0) |
| *SBT_30* | ≤17.7 | 0.66 (0.55-0.76) | 0.018 | 76.5 (50.1-93.2) | 60.3 (47.2-72.4) | 1.9 (1.3-2.9) | 0.4 (0.2-0.9) |
| **ΔEELI (ml)** | | | | | | | |
| *SBT_0* | ≤-456 | 0.77 (0.66-0.86) | <0.001 | 52.9 (27.8-77.0) | 90.5 (80.4-96.4) | 5.6 (2.3-13.4) | 0.5 (0.3-0.9) |
| *SBT_30* | ≤-743 | 0.77 (0.66-0.85) | <0.001 | 47.1 (23.0-72.2) | 92.1 (82.4-97.4) | 5.9 (2.2-15.8) | 0.6 (0.4-0.9) |
| **Inhomogeneity index (%)** | | | | | | | |
| *Baseline* | >60.6 | 0.74 (0.63-0.84) | <0.001 | 70.6 (44.0-89.7) | 76.2 (63.8-86.0) | 3.0 (1.7-5.1) | 0.4 (0.2-0.8) |
| *SBT_0* | >87.2 | 0.74 (0.63-0.83) | 0.001 | 52.9 (27.8-77.0) | 87.3 (76.5-94.4) | 4.2 (1.9-9.2) | 0.5 (0.3-0.9) |
| *SBT_30* | >58.0 | 0.75 (0.64-0.84) | <0.001 | 88.2 (63.6-98.5) | 58.7 (45.6-71.0) | 2.1 (1.5-3.0) | 0.2 (0.1-0.7) |
| **RR/Vt (breaths/min/L)** | | | | | | | |
| *Baseline* | >31 | 0.57 (0.45-0.68) | 0.386 | 70.6 (44.0-89.7) | 47.6 (34.9-60.6) | 1.3 (0.9-2.0) | 0.6 (0.3-1.3) |
| *SBT_0* | >86 | 0.80 (0.70-0.88) | <0.001 | 70.6 (44.0-89.7) | 77.8 (65.5-87.3) | 3.2 (1.8-5.5) | 0.4 (0.2-0.8) |
| *SBT_30* | >62 | 079 (0.68-0.87) | <0.001 | 88.2 (63.6-98.5) | 58.7 (45.6-71.0) | 2.1 (1.5-3.0) | 0.2 (0.1-0.7) |
| **PaO_2_/FiO_2_ (mmHg)** | | | | | | | |
| *Baseline* | ≤220 | 0.58 (0.46-0.69) | 0.414 | 35.3 (14.2-61.7) | 90.2 (79.8-96.3) | 3.6 (1.3-9.7) | 0.7 (0.5-10) |

ΔVt%, variation of tidal volume from baseline expressed in percentage; ΔEELI, variation of End-Expiratory Lung Impedance from baseline; RR/Vt, ratio between respiratory rate and tidal volume; PaO_2_/FiO_2_, ratio between arterial partial pressure and inspired fraction of oxygen; SBT, Spontaneous Breathing Trial; SBT_0, first 5 minutes of the SBT; SBT_30, last 5 minutes of the SBT; AUC, Area Under the Curve; 95% CI, 95% Confidence Interval; LR+, Positive Likelihood Ratio; LR-, Negative Likelihood Ratio.

**Table S3. Arterial Blood Gases and RR/Vt in patients with extubation success and failure**

|  | **Baseline** | **SBT_0** | **SBT_30** | **SB_0** | **SB_30** |
| --- | --- | --- | --- | --- | --- |
| **pH** | | | | | |
| *Extubation success (n=39)* | 7.45 [7.41; 7.48] | // | 7.43 [7.39; 7.46] | // | 7.43 [7.40; 7.48] |
| *Extubation failure (n=22)* | 7.42 [7.40; 7.45] | // | 7.42 [7.39; 7.46] | // | 7.42 [7.37; 7.46] |
| **Extubation success vs. failure** | p=0.397 |  | p=0.453 |  | p=0.179 |
| **PaCO_2_ (mmHg)** | | | | | |
| *Extubation success (n=39)* | 38.5 [34.0; 44.3] | // | 39.0 [35.1; 46.0] | // | 39.3 [36.7; 45.6] |
| *Extubation failure (n=22)* | 39.3 [38.0; 47.3] | // | 41.7 [36.5; 46.4] | // | 39.5 [36.0; 46.6] |
| **Extubation success vs. failure** | p=0.176 |  | p=0.467 |  | p=0.757 |
| **PaO_2_/FiO_2_ (mmHg)** | | | | | |
| *Extubation success (n=39)* | 274 [243; 303] | // | 253 [225; 295] | // | 222 [203; 281] |
| *Extubation failure (n=22)* | 270 [226; 313] | // | 246 [220; 293] | // | 213 [163; 253] |
| **Extubation success vs. failure** | p=0.932 |  | p=0.636 |  | p=0.139 |
| **RR/Vt (breaths/min/L)** | | | | | |
| *Extubation success (n=39)* | 35 [25; 60] | 59 [36; 84] | 59 [33; 94] | 49 [35; 68] | 58 [44; 84] |
| *Extubation failure (n=22)* | 38 [27; 56] | 57 [42; 86] | 61 [34; 92] | 59 [49; 96] | 77 [40; 116] |
| **Extubation success vs. failure** | p=0.712 | p=0.704 | p=0.597 | p=0.059 | p=0.185 |

RR/Vt, respiratory rate to tidal volume ratio; SBT, Spontaneous Breathing Trial; SBT_0, first 5 minutes after the beginning of the SBT; SBT_30, last 5 minutes of the SBT; SB_0, first 5 minutes of the spontaneous breathing; SB_30, last 5 minutes of the spontaneous breathing; PaCO_2_, arterial partial pressure of carbon dioxide; PaO_2_/FiO_2_, ratio between partial pressure and inspired fraction of oxygen.

**Table S4. Receiving Operating Curves of ΔVt%, ΔEELI, inhomogeneity index, RR/Vt and PaO_2_/FiO_2_ for extubation failure prediction.**

| **Parameter** | **Youden index** | **AUC (95% CI)** | **P value** | **Sensitivity (95% CI)** | **Specificity (95% CI)** | **LR+ (95% CI)** | **LR- (95% CI)** |  |
| --- | --- | --- | --- | --- | --- | --- | --- | --- |
| **ΔVt% (%)** | | | | | | | | |
| *SBT_0* | ≤-18.2 | 0.63 (0.50-0.75) | 0.071 | 68.2 (45.1-86.1) | 65.9 (49.4-79.9) | 2.0 (1.2-3.3) | 0.5 (0.3-0.9) |  |
| *SBT_30* | ≤-11.9 | 0.69 (0.56-0.80) | 0.010 | 77.3 (54.6-92.2) | 63.4 (46.9-77.9) | 2.1 (1.3-3.4) | 0.4 (0.2-0.8) |  |
| *SB_0* | ≤-17.2 | 0.70 (0.57-0.81) | 0.006 | 54.6 (32.2-75.6) | 78.1 (62.4-89.4) | 2.9 (1.5-5.6) | 0.5 (0.3-0.8) |  |
| *SB_30* | ≤-14.1 | 0.66 (0.53-0.77) | 0.030 | 72.7 (49.8-89.3) | 58.5 (42.1-73.7) | 1.8 (1.1-2.7) | 0.5 (0.1-1.0) |  |
| **ΔEELI (ml)** | | | | | | | | |
| *SBT_0* | ≤91 | 0.55 (0.42-0.67) | 0.538 | 100.0 (84.6-100.0) | 17.1 (7.2-32.1) | 1.2 (1.0-1.4) | 0.0 (0.0-0.0) |  |
| *SBT_30* | ≤-55 | 0.60 (0.46-0.71) | 0.220 | 81.8 (59.7-94.8) | 48.8 (32.9-64.9) | 1.6 (1.1-2.3) | 0.4 (0.1-1.0) |  |
| *SB_0* | ≤-317 | 0.51 (0.38-0.64) | 0.899 | 45.5 (24.4-67.8) | 65.9 (49.4-79.9) | 1.3 (0.7-2.5) | 0.8 (0.5-1.3) |  |
| *SB_30* | ≤-206 | 0.62 (0.49-0.74) | 0.110 | 63.6 (40.7-82.8) | 63.4 (46.9-77.9) | 1.7 (1.0-2.9) | 0.6 (0.3-1.0) |  |
| **Inhomogeneity index (%)** | | | | | | | | |
| *Baseline* | >44.4 | 0.61 (0.47-0.73) | 0.138 | 90.9 (70.8-98.9) | 41.5 (26.3-57.9) | 1.6 (1.2-2.1) | 0.2 (0.1-0.9) |  |
| *SBT_0* | >59.2 | 0.68 (0.55-0.79) | 0.007 | 68.2 (45.1-86.1) | 68.3 (51.9-81.9) | 2.2 (1.3-3.7) | 0.5 (0.2-0.9) |  |
| *SBT_30* | >43.6 | 0.63 (0.50-0.75) | 0.070 | 100.0 (84.6-100.0) | 31.7 (18.1-48.1) | 1.5 (1.2-1.8) | 0.0 (0.0-0.0) |  |
| *SB_0* | >58.8 | 0.66 (0.53-0.78) | 0.027 | 63.6 (40.7-82.8) | 73.2 (57.1-85.8) | 2.4 (1.3-4.3) | 0.5 (0.3-0.9) |  |
| *SB_30* | >52.7 | 0.67 (0.54-0.79) | 0.012 | 86.4 (65.1-97.1) | 51.2 (35.1-67.1) | 1.8 (1.2-2.5) | 0.3 (0.1-0.8) |  |
| **RR/Vt (breaths/min/L)** | | | | | | | | |
| *Baseline* | ≤62 | 0.53 (0.40-0.66) | 0.681 | 90.9 (70.8-98.9) | 24.4 (12.4-40.3) | 1.2 (1.0-1.5) | 0.4 (0.1-1.6) |  |
| *SBT_0* | >39 | 0.54 (0.40-0.66) | 0.650 | 86.4 (65.1-97.1) | 36.6 (22.1-53.1) | 1.4 (1.0-1.8) | 0.4 (0.1-1.1) |  |
| *SBT_30* | >38 | 0.55 (0.42-0.67) | 0.533 | 72.7 (49.8-89.3) | 41.5 (26.3-57.9) | 1.2 (0.9-1.8) | 0.7 (0.3-1.4) |  |
| *SB_0* | >51 | 0.65 (0.52-0.77) | 0.045 | 77.3 (54.6-92.2) | 56.1 (39.7-71.5) | 1.8 (1.2-2.7) | 0.4 (0.2-0.9) |  |
| *SB_30* | >66 | 0.61 (0.48-0.73) | 0.182 | 59.1 (36.4-79.3) | 68.3 (51.9-81.9) | 1.9 (1.1-3.3) | 0.6 (0.3-1.0) |  |
| **PaO_2_/FiO_2_ (mmHg)** | | | | | | | | |
| *Baseline* | ≤231 | 0.52 (0.39-0.64) | 0.842 | 31.2 (13.9-54.9) | 82.9 (67.9-92.8) | 1.9 (0.7-4.6) | 0.8 (0.6-1.1) |  |
| *SBT_30* | ≤234 | 0.55 (0.42-0.67) | 0.555 | 50.0 (28.2-71.8) | 70.7 (54.5-83.9) | 1.7 (0.9-3.2) | 0.7 (0.4-1.1) |  |
| *SB_30* | ≤185 | 0.62 (0.49-0.74) | 0.122 | 40.9 (20.7-63.6) | 87.8 (73.8-95.9) | 3.4 (1.3-8.8) | 0.7 (0.5-1.0) |  |

ΔVt%, variation of tidal volume from baseline expressed in percentage; ΔEELI, variation of End-Expiratory Lung Impedance from baseline; RR/Vt, ratio between respiratory rate and tidal volume; PaO_2_/FiO_2_, ratio between arterial partial pressure and inspired fraction of oxygen; SBT, Spontaneous Breathing Trial; SBT_0, first 5 minutes of the SBT; SBT_30, last 5 minutes of the SBT; SB_0, first 5 minutes of the spontaneous breathing; SB_30, last 5 minutes of the spontaneous breathing; AUC, Area Under the Curve; 95% CI, 95% Confidence Interval; LR+, Positive Likelihood Ratio; LR-, Negative Likelihood Ratio.

**Table S5. EIT data in patients with “rescue” NIV success and failure.**

|  | **Baseline** | **SBT_0** | **SBT_30** | **SB_0** | **SB_30** |
| --- | --- | --- | --- | --- | --- |
| **ΔVt% (%)** | | | | | |
| *NIV success (n=11)* | 0 [0; 0] | -20 [-31; -11] | -14 [-40; -9] | -10 [-20; 4] | -17 [-24; -5] |
| *NIV failure (n=8)* | 0 [0; 0] | -36 [-42; -10] | -32 [-53; -13] | -37 [-54; 21] | -42 [-63; -14] |
| **NIV success vs. failure** | *p>0.999* | *p=0.238* | *p=0.351* | *p=0.075* | *p=0.395* |
| **ΔEELI (ml)** | | | | | |
| *NIV success (n=11)* | 0 [0; 0] | -94 [-221; 53] | -239 [-284; 98] | -29 [-375; 399] | -4 [-434; 355] |
| *NIV failure (n=8)* | 0 [0; 0] | -178 [-614; -53] | -194 [-677; -60] | -269 [-820; 79] | -571 [-948; 228] |
| **NIV success vs. failure** | *p>0.999* | *p=0.109* | *p=0.657* | *p=0.177* | *p=0.051* |
| **Inhomogeneity index (%)** | | | | | |
| *NIV success (n=11)* | 54 [45; 71] | 59 [48; 74] | 58 [46; 64] | 54 [45; 79] | 59 [46; 67] |
| *NIV failure (n=8)* | 55 [49; 60] | 67 [61; 92] | 66 [56; 74] | 69 [57; 76] | 69 [59; 76] |
| **NIV success vs. failure** | *p=0.840* | *p=0.177* | *p=0.238* | *p=0.395* | *p=0.310* |

SBT, Spontaneous Breathing Trial; SBT_0, first 5 minutes after the beginning of the SBT; SBT_30, last 5 minutes of the SBT; SB_0, first 5 minutes of the spontaneous breathing; SB_30, last 5 minutes of the spontaneous breathing; ΔVt%, change from baseline of the tidal volume in percentage; ΔEELI, change from baseline of the end-expiratory lung impedance.

**Table S6. EIT data in patients with respiratory and non-respiratory reasons of extubation failure.**

|  | **Baseline** | **SBT_0** | **SBT_30** | **SB_0** | **SB_30** |
| --- | --- | --- | --- | --- | --- |
| **ΔVt% (%)** | | | | | |
| *Respiratory (n=15)* | 0 [0; 0] | -23 [-40; -15] | -27 [-44; -12] | -18 [-38; 1] | -21 [-44; -14] |
| *Non-respiratory (n=7)* | 0 [0; 0] | -33 [-43; -3] | -30 [-47; 9] | -35 [-47; 3] | -35 [-62; 5] |
| **Respiratory vs. non-respiratory** | *p>0.999* | *p>0.999* | *p>0.999* | *p=0.680* | *p=0.945* |
| **ΔEELI (ml)** | | | | | |
| *Respiratory (n=15)* | 0 [0; 0] | -106 [-336; 14] | -239 [-328; 60] | -262 [-383; 188] | -220 [-383; 188] |
| *Non-respiratory (n=7)* | 0 [0; 0] | -119 [-238; 24] | -94 [-356; -55] | -211 [-518; 133] | -384 [-931; 53] |
| **Respiratory vs. non-respiratory** | *p>0.999* | *p=0.945* | *p=0.783* | *p=0.490* | *p=0.298* |
| **Inhomogeneity index (%)** | | | | | |
| *Respiratory (n=15)* | 59 [48; 62] | 67 [53; 91] | 62 [53; 82] | 60 [48; 81] | 61 [53; 86] |
| *Non-respiratory (n=7)* | 51 [45; 53] | 64 [55; 68] | 62 [54; 70] | 66 [56; 72] | 66 [61; 73] |
| **Respiratory vs. non-respiratory** | *p=0.332* | *p=0.783* | *p=0.945* | *p>0.999* | *p=0.783* |

SBT, Spontaneous Breathing Trial; SBT_0, first 5 minutes after the beginning of the SBT; SBT_30, last 5 minutes of the SBT; SB_0, first 5 minutes of the spontaneous breathing; SB_30, last 5 minutes of the spontaneous breathing; ΔVt%, change from baseline of the tidal volume in percentage; ΔEELI, change from baseline of the end-expiratory lung impedance.

**Figure S1. Flow diagram of screened and enrolled patients**

Excluded from data analysis (n=2)

- Incorrect inclusion (n=2)

- Protocol interruption (n=0)

Included in data analysis (n=78)

Enrolled in the study (n=80)

Excluded (n=65)

- Pneumothorax or pulmonary emphysema (n=15)

- Recent thoracic surgery (n=12)

- Included in other research protocol (n=36)

- Declined to participate (n=2)

Assessed for eligibility (n=145)

Admitted in the ICUs (n=1555)

Not eligible (n=1410)

- Not receiving invasive Mechanical Ventilation (iMV) (n=436)

- Receiving iMV <48 hours (n=478)

- Not “at risk” for post-extubation respiratory failure (n=453)

- Age <18 years/old (n=43)
